# Cardiac-derived extracellular matrix: A decellularization protocol for heart regeneration

**DOI:** 10.1371/journal.pone.0276224

**Published:** 2022-10-19

**Authors:** Immacolata Belviso, Anna Maria Sacco, Domenico Cozzolino, Daria Nurzynska, Franca Di Meglio, Clotilde Castaldo, Veronica Romano

**Affiliations:** 1 Department of Public Health, University of Naples Federico II, Naples, Italy; 2 Department of Medicine, Surgery and Dentistry, Scuola Medica Salernitana, University of Salerno, Baronissi, Italy; IRCCS San Raffaele Pisana, ITALY

## Abstract

Extracellular matrix (ECM) is a fundamental component of the heart, guiding vital cellular processes during organ homeostasis. Most cardiovascular diseases lead to a remarkable remodeling of the ECM, accompanied by the formation of a fibrotic tissue that heavily compromises the heart function. Effective therapies for managing fibrosis and promoting physiological ECM repair are not yet available. The production of a decellularized extracellular matrix (d-ECM) serving as a three-dimensional and bioactive scaffold able to modulate cellular behavior and activities is considered crucial to achieve a successful regeneration. The protocol represents a step-by-step method to obtain a decellularized cardiac matrix through the combination of sodium dodecyl sulphate (SDS) and Triton X-100. Briefly, cardiac samples obtained from left ventricles of explanted, pathological human hearts were dissected and washed to remove residual body fluids. Samples were then snap-frozen and sliced by a cryostat into 350 μm thick sections. The sections obtained were decellularized using a solution containing 1% Triton X-100 and 1% SDS in combination, for 24 hours, until observing the color change from brownish-red to translucent-white. As a result, the protocol shows efficiency in preserving ECM architecture and protein composition during the whole process, suggesting that it is worthwhile, highly reproducible and produces a well- preserved decellularized extracellular matrix from cardiac samples. Notwithstanding, some limitations need to be addressed, such as the risk for microbial contamination and the unpredictable trend of the protocol when applied to decellularize samples other than myocardium, vessels, or skin. These issues require antibiotics mixture supplement during the procedure followed by UV sterilization, and appropriate adjustments for a tissue-specific utilization, respectively. The protocol is intended to produce a cardiac d-ECM for cell settlement, representing the ideal scaffold for tissue engineering purposes.

## Introduction

Regenerative medicine and cardiac tissue engineering are turning their attention on the application of decellularized extracellular matrix (d-ECM) to remodel, replace and regenerate damaged or impaired heart [1]. The production of a three-dimensional scaffold owning features suitable to modulate cellular behavior and activities for an effective regeneration is pivotal, and it is actually focused on the share of three major components: scaffold, cells and biosignals [2]. Extracellular matrix (ECM) is a three-dimensional network containing macromolecules such as structural proteins like collagen, enzymes, growth factors and complex polysaccharides, synthesized by cells to create a specialized microenvironment for their support, able to influence their biology and conferring specific physical, chemical, and mechanical properties to the tissue [3–9]. Cardiac ECM is typically composed of glycosylated proteins and polysaccharides, including fiber proteins, laminin, tenascin, and fibronectin, each one playing a specific role. The native ECM synthetized by resident cells represents a biological and naturally functionalized support, responsible for cell-cell and cell-microenvironment crosstalk and interaction, and affecting the fate of cell migration, differentiation, and apoptosis [10]. The high complexity of natural ECM makes hard to reproduce it synthetically. Hence, many researchers focused on the development of a biological ECM suitable for regenerative medicine by decellularizing tissues, preserving as much as possible their complex architecture and removing immunogenic components responsible for immune rejection [11]. ECM components are widely used; they are commonly isolated and purified from natural tissues and employed, as a single component or mixtures, for cardiac regeneration in the form of injectable hydrogels, or as cardiac patches directly implanted into the infarcted myocardium, sometimes repopulated with selected cells, and functionalized introducing bioactive compounds [12–14]. Although purified ECM components are considered a promising tool for clinical application in the treatment of ischemic heart disease, a major issue remains, as their structural integrity and biological activity are partially or completely lost due to their purification and isolation processes, and they are not best performing under biological and biomechanical profile when compared to native cardiac ECM [15]. A novel natural ECM, composed of decellularized myocardium is intensively researched for cardiac tissue engineering applications, as it may represent the ideal scaffold, offering a preserved composition and architecture along with a biological activity suitable for their further repopulation with cells [16–18]. ECM density, morphology and thickness can vary from organ to organ, thus the set-up of decellularization protocol should be specific for each type of tissue [19]. Several protocols describe a sequential or a combinatorial effect of enzymatic, chemical, and physical techniques, although physical ones are not used alone, but rather as a support to chemical and enzymatic techniques to boost tissue decellularization [20]. The ideal decellularization of cardiac tissues requires the complete removal of cells, leaving pristine structural, biochemical, and mechanical properties of the ECM [21]. Several techniques have been tuned to optimize the decellularization of cardiac tissue, including the use of physical, biological, or chemical methods [22, 23]. The most popular physical methods for decellularization are freeze-thawing cycles and the application of mechanical forces to disrupt cell membranes. Even though they are effective in causing a complete lysis of cells, they could result in damage to the ECM, due to ice crystals formation and mechanical stress, respectively. Biological procedures leverage enzymatic reactions for tissue decellularization. The enzymes commonly used are nucleases and proteases, worthy to degrade RNA and DNA by cleaving off specific bonds within or at the end of nucleic acids, or by denaturing proteins. The application of this method usually causes a cellular and even ECM disruption [24]. Chemical methods are essentially based on the use of non-ionic or ionic detergents for cellular removal. Chemical techniques are also used in combination with physical methods to reach a whole tissue decellularization. The most used non-ionic detergent is Triton X-100 which targets lipid-lipid and lipid-protein interactions, leaving protein-protein interactions intact [25, 26]; Sodium Dodecyl Sulphate (SDS) is an example of ionic detergent widely used in decellularization protocols, effective to remove cells by solubilizing their membranes. Prolonged exposures to SDS can cause protein denaturation, and ECM structure alteration [27]. To prevent ECM from chemical damages, it could be recommendable to shorten the incubation time to obtain a decellularized matrix with a well-preserved architecture [28]. Here, we describe a novel protocol to produce decellularized ECM from human heart, through the combinatorial effect of SDS and Triton X-100, with a short incubation time of the sample, aiming at minimizing the disruption of the matrix.

The proposed protocol has been successfully used, with minor adjustments, to prepare scaffolds from human skin, showing a high cytocompatibility, supporting cell adhesion, migration, differentiation, and survival *in vitro*, and also tested in stretch bioreactor to develop more mature and performing 3D supports for cardiac regeneration [28–31]. Although the procedure allows to obtain a d-ECM with minimal mechanical and chemical disruption, during the entire process microbial contaminations could affect the biological quality of the decellularized tissue, therefore, in addition to the use of antibiotics, a sterilization cycle under UV is mandatory prior to use [32]. However, it should be noted that the procedure has been optimized for decellularization of skin, myocardium, and vessels only, thus, it is likely that the trend of decellularization is different based on the tissue source and needs further adaptation of the protocol proposed in this study [11].

## Materials and methods

The protocol described in this peer-reviewed article is published on protocols.io, **https://dx.doi.org/10.17504/protocols.io.4r3l2o22xv1y/v2** and is included for printing as S1 File with this article. All cardiac tissue samples were obtained from explanted hearts of patients (n = 10, mean age 49.5 ± 4.7) undergoing heart transplantation because of end‐stage heart failure associated with ischemic cardiomyopathy.

Methods for quantitative measurement of DNA content, quantitative measurement of collagen and sGAG, immunohistochemistry and Real-time PCR are described in details in supporting info (S1 Text).

### Ethics statement

Patients provided written informed consent for use of heart tissue for experimental studies and specimens were collected, without patient identifiers, following protocols approved by Comitato Etico University of Naples Federico II.

### Results

The proposed protocol provides a fast and reproducible method to obtain decellularized extracellular matrix from cardiac tissue as reported in Table 1.

**Table 1:** Protocol steps scheme.

DAY 1			DAY 2			DAY 3		
T	TIME	BUFFER/REAGENT	T	TIME	BUFFER/REAGENT	T	TIME	BUFFER/REAGENT
RT	24 hrs	1% SDS, 1% Triton X-100 in double-distilled water	RT	24 hrs	0.25 μg/ml Amphotericin B, 100 U/ml Penicillin, 50 U/ml Streptomycin in 1x PBS	RT	30 min	Double-distilled water

The combination of Triton X-100 and SDS and the short time of exposure during the decellularization procedure allows the preservation of the three-dimensional architecture and protein composition of the samples.

**Fig 1:**
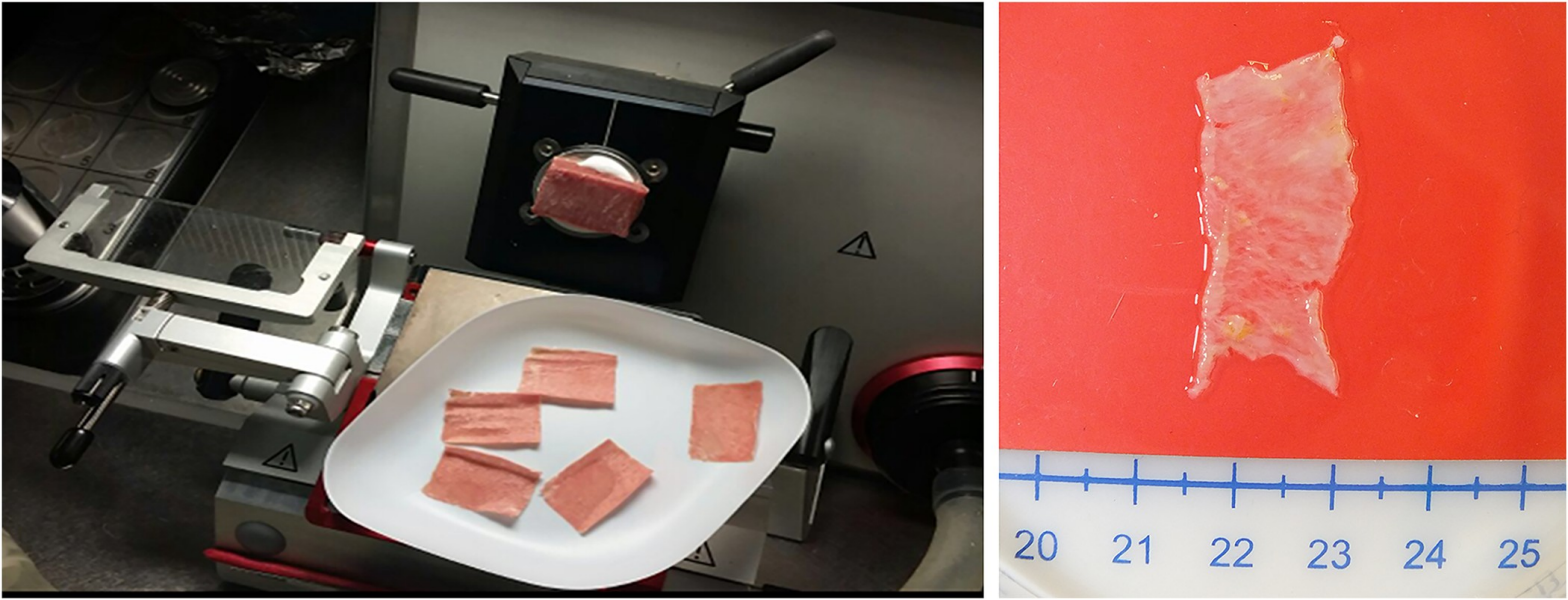
Sections of left ventricles sliced by cryostat. Macroscopic observation showing the color change from brownish-red to translucent-white.

At the end of the decellularization procedure the macroscopic observation of myocardial sections, shows an intact tissue architecture and the typical color shift from brownish-red to translucent-white, as a result of a complete decellularization (Fig 1).

**Fig 2:**
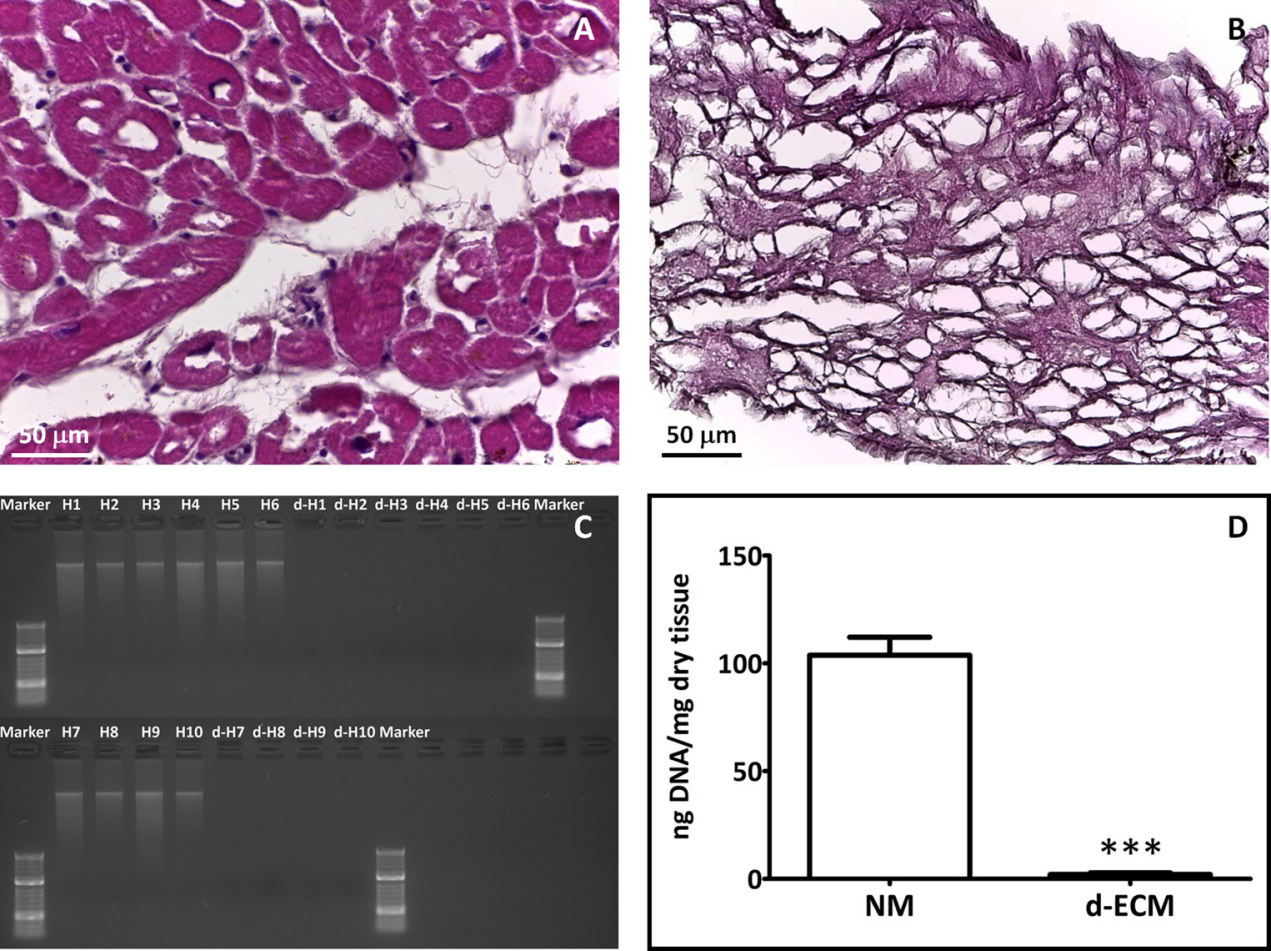
Evaluation of decellularization procedure effectiveness. Representative images of Hematoxylin and Eosin staining (HE) on cryosections of native myocardium (A) or of d-ECM (B). Scale bar length is 50 μm. C: Agarose gel electrophoresis analysis of dsDNA isolated from native myocardium and d-ECM. D: Graphical representation showing a DNA content in native myocardium (NM) of 103.8 ± 8.34 and in d-ECM of 2.049 ± 0.7186 ng per mg of dry tissue. Each value expresses the mean of 10 samples (n = 10) ± SEM (*** p < 0.0001).

Hematoxylin and Eosin (HE) staining reveals the absence of any cellular debris or nuclei in d-ECM (Fig 2B) when compared to the native tissue (Fig 2A), proving the effectiveness of the procedure. Appropriately decellularized tissue is defined by the literature [21] as having a DNA content below 50 ng/mg tissue. Analyzed d-ECM according to AllPrep DNA/RNA Mini Kit (Qiagen, Hilden, Germany) instructions, showed a removal of dsDNA of 97.90 ± 0.86% confirming the efficacy of the decellularization (Fig 2D). The absence of residual DNA fragments was also checked out by the electrophoresis on agarose gel, showing no DNA band for d-ECM (Fig 2C).

Minimal data set are available in Supporting information (S1 Data).

Additionally, the content and the distribution of the specific ECM proteins appears unaltered, as showed by the histochemical analysis.

**Fig 3:**
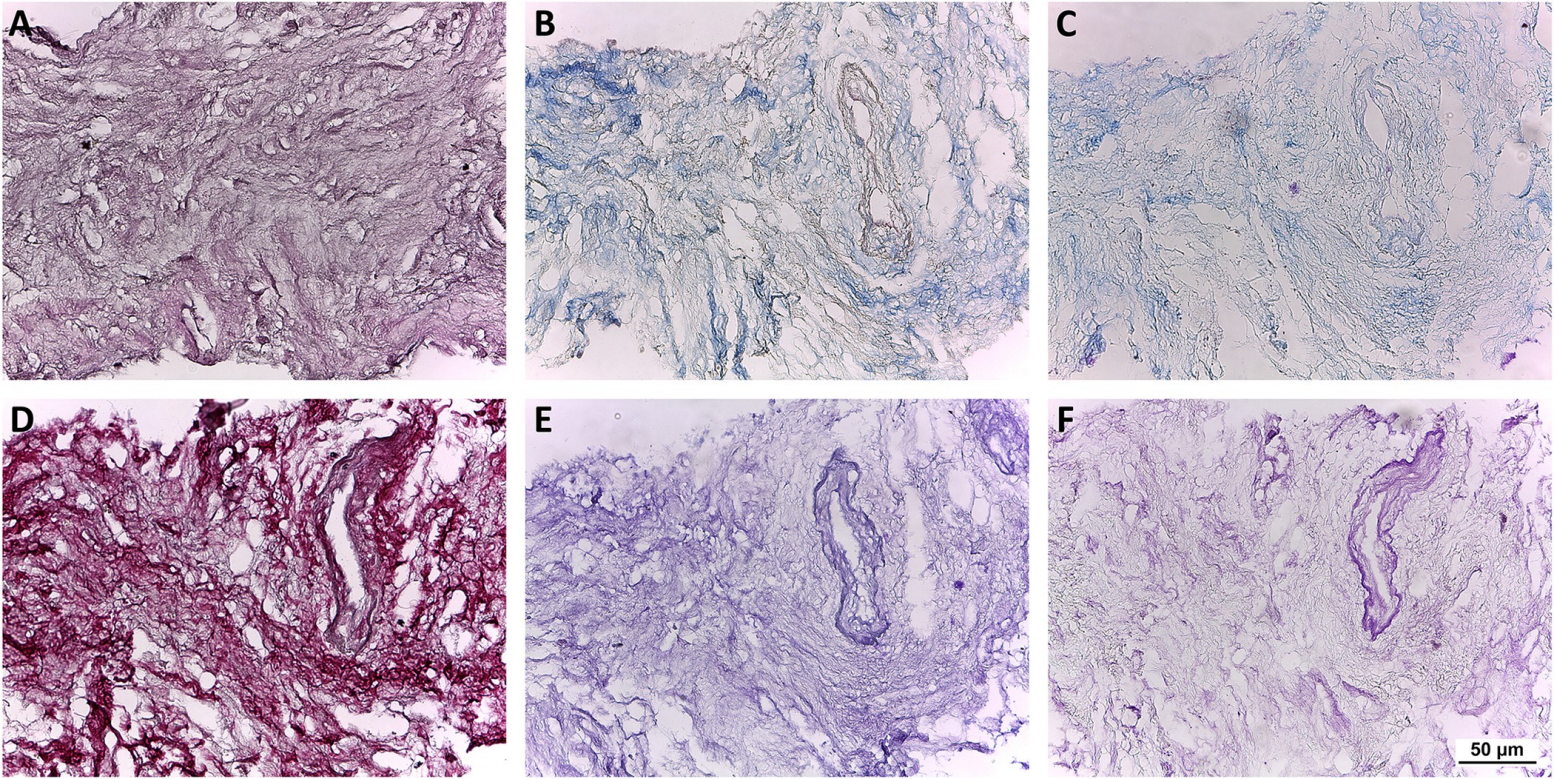
Evaluation of the d-ECM architecture and composition by histochemistry. Representative images of Hematoxylin and Eosin staining (A) showing no residual nuclei, Masson’s and Mallory’s Trichrome (B and C, respectively), Sirius Red (D), PAS Morel-Maronger modified (E) and Gomori’s paraldehyde-fuchsin (F) stainings on cryosections of d-ECM showing collagen fibers stained blue (B-C) or red (D), glycoproteins stained violet (E) and elastic fibers stained pink (F). Scale bar length is 50 μm.

Masson’s and Mallory’s trichrome and Sirius Red stainings showed the preservation of collagen fibers as well, while PAS Morel‐Maronger and Gomori’s stainings make evident the retention of non‐collagenous proteins and elastic fibers, respectively (Fig 3).

Additional images are available in Supporting information (S1 Fig).

**Fig 4:**
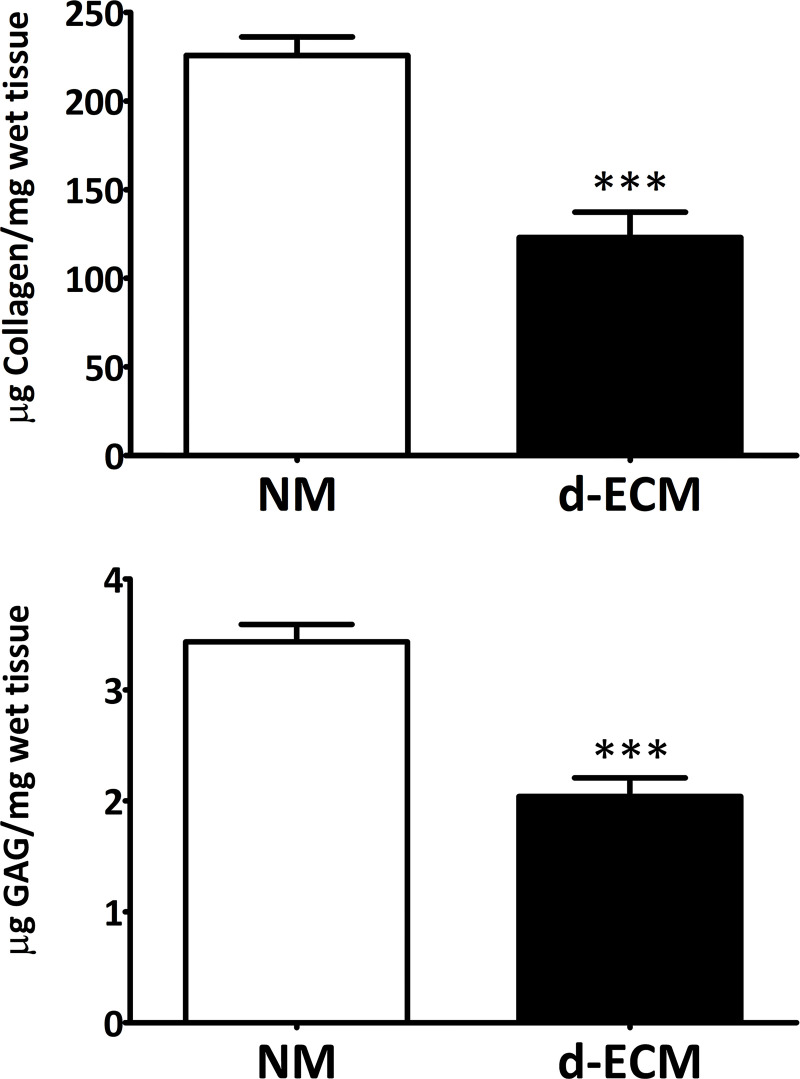
Quantitative measurements of collagen and sGAG in native myocardium and d-ECM. Data showing the retention of collagen (123.0 ± 14.33 μg/mg wet tissue) and sGAG (2.039 ± 0.1681 μg/mg wet tissue) in d-ECM compared with native myocardium (225.8 ± 10.41 μg/mg wet tissue; 3.433 ± 0.1572 μg/mg wet tissue). Each value expresses the mean of 10 samples (n = 10) ± SEM (*** p ≤ 0.0001).

The content of collagen and sulfated glycosaminoglycan (sGAG) was investigated by Sircol or Blyscan quantitative dyebinding assay, respectively. The quantitative measurements of collagen and sGAG in d‐ECM demonstrates a high retention of both components. Accordingly, collagen residual content resulted 56.55 ± 8.07%, while sGAG showed a retention of 59.30 ± 3.85% (Fig 4).

Minimal data sets are available in supporting information (S2 and S3 Data).

**Fig 5:**
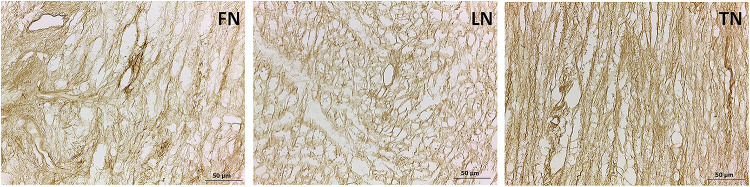
Evaluation by immunohistochemistry of structural protein retention in d-ECM. Representative images of fibronectin (FN), laminin (LN) and tenascin (TN) distribution in cardiac d-ECM. Scale bar length is 50 μm.

Immunohistochemistry also highlights the retention of fibronectin, laminin, and tenascin in the decellularized extracellular cardiac matrix, supporting that the decellularizing method is conservative for the structural proteins as well (Fig 5).

Additional images are available in Supporting information (S2 Fig).

**Fig 6:**
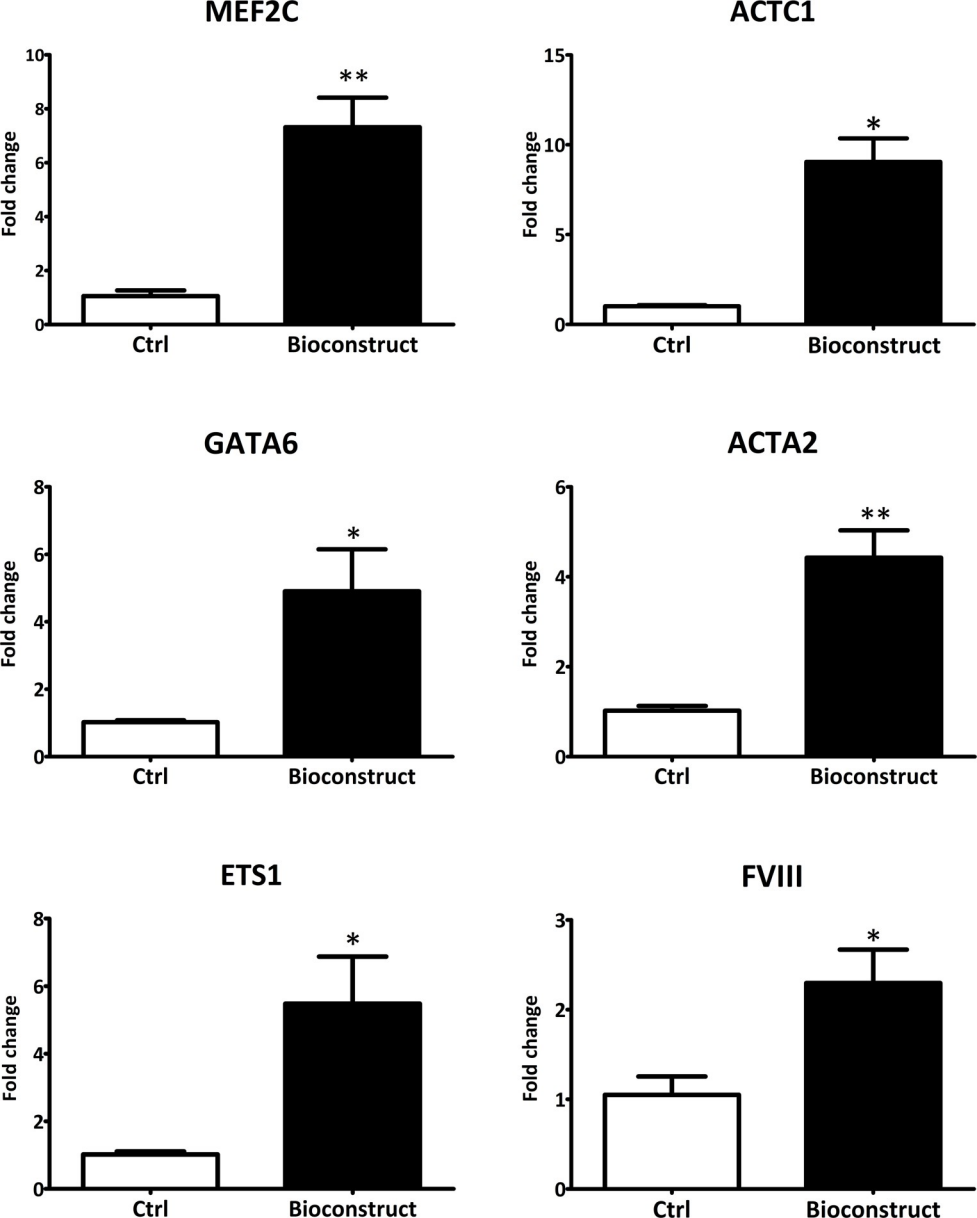
Gene expression analysis of cardiac cell markers in hCPCs culterd on d‐ECM or on plastic. Real-time PCR analysis for cardiac gene expression showed an upregulated transcription of cardiomyocyte markers MEF2C and ACTC1 (7.320 ± 1.096 and 9.053 ± 1.305), smooth muscle cell markers GATA6 and ACTA2 (4.903 ± 1.245 and 4.423 ± 0.6064) and endothelial cell markers ETS1 and FVIII (5.480 ± 1.394 and 2.297 ± 0.3720) (*p ≤ 0.05; **p ≤ 0.001).

Real-Time PCR revealed that d‐ECM obtained with the described protocol supports cell living through and differentiation towards cardiac lineages. Bioconstructs were prepared by culturing human cardiac progenitor cells (hCPCs) isolated from adult human hearts (n = 10) on d-ECM scaffolds (n = 3) previously sterilized and rehydrated with culture medium. After 21 days of culture hCPCs were detached from the d-ECM and analyzed by Real-time PCR. From the analysis emerged that hCPCs culterd on d‐ECM showed a statistically significant up-regulation of genes involved in the differentiation towards cardiomyocytes (MEF2C and ACTC1), endothelial (ETS1 and FVIII) and smooth muscle cells (GATA6 and ACTA2) with respect to hCPCs cultered on plastic under the same conditions (Fig 6).

Minimal data set are available in Supporting information (S4 Data).

The natural d-ECM obtained by applying this protocol shows a huge potential in cardiac tissue engineering. The scaffold could provide a suitable microenvironment along with biological signals for damaged heart, prompting the tissue reconstruction upon implantation. The preservation of qualitative and quantitative integrity of the ECM after the decellularization method offers a good basis for a facilitating recellularization, reintroducing cells into the specific compartment of the scaffold, mimicking normal tissue structure.

For the near future, the d-ECM produced by the method proposed could be concretely considered for pre-clinical and clinical applications, allowing the construction of patches for cardiac repair.

## Supporting information

S1 File Step-by-step protocol also available on protocols.io. (PDF)

S1 Fig Histochemistry on native and decellularized criosections. Representative images of Hematoxylin and Eosin, Masson’s and Mallory’s Trichrome, Sirius Red, PAS Morel-Maronger modified and Gomori’s paraldehyde-fuchsin stainings on cryosection sets of d-ECM (S1-S7) compared to liver sections as negative control (NC) and native heart sections as positive control (PC1-PC2). Scale bar length is 50 μm.
(TIF)

S2 Fig Immunohistochemistry of ECM structural proteins. Representative images of fibronectin, laminin and tenascin distribution in cardiac d-ECM. Scale bar length is 50 μm.
(TIF)

S1 Data DNA content minimal data set. (XLSX)

S2 Data Collagen content minimal data set. (XLSX)

S3 Data sGAG content minimal data set. (XLSX)

S4 Data Real-time minimal data set. (XLSX)

S1 Table Primer sequences of genes analyzed by real-time PCR. (DOCX)

S1 Text Methods supporting file. (DOCX)
