# Supplementary material for: Cardiac-derived extracellular matrix: A decellularization protocol for heart regeneration
Source: PLoS One. 2022 Oct 19;17(10):e0276224. doi: 10.1371/journal.pone.0276224 (PMC9581349; doi:10.1371/journal.pone.0276224)
Supplement: S1 File — (PDF) [file pone.0276224.s001.pdf]

Human myocardium decellularization V. (cec9taz6)

cec9taz6

Immacolata Belviso[1], Anna Maria Sacco[1], Domenico Cozzolino[1], Daria Nurzynska[2], Franca Di Meglio[1], clotilde.castaldo [1], Veronica Romano[1]

[1]Department of Public Health, University of Naples Federico II, Naples, Italy;
[2]Department of Medicine, Surgery and Dentistry, Scuola Medica Salernitana, University of Salerno, Baronissi, Italy

1 Works for me

Reserved DOI:
10.17504/protocols.io.4r3l2o22xv1y/v2

clotilde.castaldo

ABSTRACT

The protocol represents a step-by-step method to obtain a decellularized cardiac matrix through the combination of sodium dodecyl sulphate (SDS) and Triton X-100. Briefly, cardiac samples obtained from left ventricles of explanted, pathological human hearts were dissected and washed to remove residual body fluids. Samples were then snap-frozen and sliced by a cryostat into 350 µm thick sections. The sections obtained were decellularized using a solution containing 1% Triton X-100 and 1% SDS in combination, for 24 hours, until observing the color change from brownish-red to translucent-white. As a result, the protocol shows efficiency in preserving extracellular matrix architecture and protein composition during the whole process, suggesting that it is worthwhile, highly reproducible and produces a well- preserved decellularized extracellular matrix from cardiac samples.

PROTOCOL INFO

Immacolata Belviso, Anna Maria Sacco, Domenico Cozzolino, Daria Nurzynska, Franca Di Meglio, clotilde.castaldo , Veronica Romano . Human myocardium decellularization. **protocols.io**
https://protocols.io/view/human-myocardium-decellularization-cec9taz6
Version created by clotilde.castaldo

CREATED

Jul 27, 2022

LAST MODIFIED

Jul 28, 2022

PROTOCOL INTEGER ID

67713

Preparation of decellularizing solution 50m

1 **Preparation of 600 mL of decellularizing solution** 50m

1.1 Prepare 300 mL of 2% Triton X-100 solution by measuring 294 mL of double-distilled water in a graduated cylinder and transferring it to a 500 mL beaker. 2m

1.2 2m

Add 6 mL of Triton X-100 to the beaker containing the double-distilled water using a serological pipette.

Triton X-100 **Sigma Aldrich Catalog #X100-1L**

It is recommended to wear personal protective devices.

1.3 15m

Heating Magnetic Stirrer

VELP SCIENTIFICA VP-F20520162

Add a stir bar into the beaker and place it on a magnetic stirrer to mix the solution until completely dissolved.

1.4 Prepare 300 mL of 2% SDS solution by measuring 275 mL of double-distilled water in a graduated cylinder and transferring it to a 500 mL beaker. 2m

1.5 5m

Explorer Pro Precision EP413
Precision balance
Ohaus 80108921

Weigh 6 g of SDS powder in a weighing boat using a spoon and an electronic balance. Transfer the powder to the beaker containing the double-distilled water.

Sodium dodecyl sulfate **Sigma Aldrich Catalog #62862**

This step shoul be performed under chemical hood wearing personal protective devices.

1.6 Add a stir bar into the beaker and place it on a magnetic stirrer to mix the solution until completely dissolved. 10m

1.7 Pour the solution in a graduated cylinder and adjust the volume to 300 mL by adding double-distilled water. 4m

1.8 5m

Pour 2% Triton X-100 and 2% SDS solutions, previously prepared, in a 1 L cylinder to obtain a total volume of 600 ml of 1% decellularizing solution. Cover with parafilm and gently mix by inversion to obtain a homogeneous solution.

Parafilm M
Thermoplastic film
Sigma-Aldrich P7793-1EA

1.9 Transfer 1% decellularizing solution in a 1 L graduated bottle using a funnel to reduce foaming. 5m
Store at +4 °C until use.

The final volume of the decellularizing solution can vary according to the number of samples to decellularize. The volume reported in the protocol is intended for 15 samples.

Preparation of 1x phosphate buffered saline (PBS) solution 29m

2 Preparation of 500 mL of 1x PBS

29m

2.1 Weigh all the salt powders in recommended amounts using an electronic balance, a spatula and a spoon. 5m

0.1 g Potassium Phosphate Monobasic

0.1 g Potassium Chloride

4.0 g Sodium Chloride

0.575 g Sodium Phosphate Dibasic

Transfer the salts into a 500 mL beaker.

Potassium Phosphate Monobasic **Contributed by users Catalog #P5655-1Kg**

Potassium Chloride **Sigma Aldrich Catalog #P9333**

Sodium Chloride **Sigma Aldrich Catalog #S7653**

Sodium Phosphate Dibasic **Sigma Aldrich Catalog #S9763-1Kg**

2.2 Take a graduated cylinder to measure **400 mL** of double-distilled water and pour it into the beaker. 2m

2.3 Add a stir bar and place the beaker on a magnetic stirrer to completely dissolve the salts. 15m

2.4 Pour the solution in a graduated cylinder and adjust the volume to 500 mL by 2m

adding double-distilled water.

2.5 Check the pH value and adjust to pH 7.4 if needed. 5m

Store at +4 °C untile use.

Preparation of antibiotic solution 5m

3 Preparation of 10 mL antibiotic solution 5m

3.1 Accurately weigh **625 µg Amphotericin B** using an electronic balance and add it to a **8 mL pen/strep mixture** . Mix vigorously until it is completely dissolved. 3m

Amphotericin B **Sigma Aldrich Catalog #Y0000005**

Penicillin-Streptomycin **Sigma Aldrich Catalog #P4333**

3.2 Pour the solution in a graduated cylinder and adjust the volume to 10 mL adding pen/strep mixture. 2m

Store at **+4 °C** until use.

Preparation of samples and decellularization procedure 1d 13h 16m

4 Preparation and decellularization of samples 1d 13h 16m

4.1 10m

Identify and wash the cardiac tissue samples obtained from explanted hearts into a plastic tray using a [M] **0.9 Mass / % volume Sodium Chloride isotonic solution** to remove any residual fluid.

Sodium Chloride Solution **Sigma Aldrich Catalog #S8776**

4.2 5m

Dissecting Board
Board for Anatomical Dissection
VWR 100498-398

Prepare a set of large surgical scissors, long forceps, fine forceps and scalpel needed to dissect the heart. Use a dissecting board with graduations to measure sample size.

4.3 ⚠ 10m

Cut unrefined samples from full-thickness left ventricle wall avoiding injured areas and wash with
[M]**0.9 Mass / % volume Sodium Chloride isotonic solution** .

4.4 ⚠ 15m

Place them on the dissecting board and cut, by a dissecting scalpel, 2 cm x 2 cm (lenght by width) fragments using the graduation on the dissection board as a reference.

Fragments should not be larger than 2 cm wide by 2 cm long.

4.5 Snap freeze at **-80 °C** . 1h

4.6 1d 13h 16m

Cryostat
Leica CM1950

Mount samples on cryostat chuck and slice them one by one to obtain **350 µm** thick sections.

It is recommended to cut at least three 350-µm-thick sections of each sample, using as a reference the same number of sections of native tissue.

4.7 Prepare and label with all the information identifying the samples a 50 mL tube for each section. Add 40 mL of decellularizing solution previously prepared go to step #1 , place one section in each tube. 20m

Make sure the tubes are appropriately locked to avoid solution leakage.

4.8 1d

Platform Rocker STR6
Orbital Shaker
Stuart Scientific L065

Place the tubes on an orbital shaker and start the procedure setting moderate speed of agitation for 24 hours, at Room temperature .

4.9 Replace the decellularizing solution in each 50 mL tube with 40 mL of 1x PBS go to step #2 and 0.2 mL of antibiotic solution go to step #3 . 30m

4.10 ⚠ 10m

Stop the agitation and check the color of the sections.

Samples should shift from the native red to translucent white.

4.11 8h

Start the agitation on the orbital shaker at a moderate speed overnight, at Room temperature .

4.12 30m

Stop the agitation. Replace the solution in each 50 mL tube with **40 mL** of double-distilled water.

4.13 Start the agitation on the orbital shaker at a moderate speed for 30 minutes at **Room temperature** . 30m

4.14 Stop the agitation. Open each tube and gently dry sections to remove the excess of double-distilled water. 30m

Sample storage 30m

5 Fix decellularized sections for histological analyses. 30m

Store at **+4 °C** in a [M]**0 Mass / % volume Sodium Chloride isotonic solution** for further cell seeding or snap-freeze at **-80 °C** for other applications.

A cycle of sterilization under UV is highly recommended before cell seeding, and d-ECM must be rehydrated with an appropriate culture medium prior to use.

Materials List

6 **Addictional materials**

EQUIPMENT	BRAND	CATALOG NUMBER	SPECIFICATION
1 L beaker	VWR	511-0318	Clean and autoclave before use
10 mL serological pipette	Falcon	357551	Sterile, polystyrene
50 mL sterile tubes	Falcon	FC-1 352070	Sterile tubes, polypropylene
10 mL graduated cylinder	VWR	612-1518	Clean and autoclave before use

1L graduated cylinder	VWR	612-1524	Clean and autoclave before use
1 L bottle	VWR	215-1596	Clean and autoclave before use
25 mL serological pipette	Falcon	357525	Sterile, polystyrene
500 mL beaker	VWR	511-0317	Clean and autoclave before use
Dissecting scalpel	VWR	233-5526	Sterile and disposable
Fine forceps	VWR	232-1317	Clean and autoclave before use
Funnel	VWR	221-1861	Clean and autoclave before use
Hexagonal weighing boats size M	Sigma-Aldrich	Z708585	Hexagonal, polystyrene, 51 mm
Hexagonal weighing boats size S	Sigma-Aldrich	Z708577	Hexagonal, polystyrene, 25 mm
Large surgical scissors	VWR	233-1211	Clean and autoclave before use
Long forceps	VWR	232-0096	Clean and autoclave before use
Pipette gun	Eppendorf	613-2795	Eppendorf Easypet® 3
Plastic tray	VWR	BELAH162620000	Corrosion-proof polypropylene
Spatula	VWR	RSGA038.210	Clean and autoclave before use
Spoon	VWR	231-1314	Clean and autoclave before use
Stir bar	VWR	442-0362	Clean and autoclave before use
