# Supplementary figures and images for: Cardiac-derived extracellular matrix: A decellularization protocol for heart regeneration
Source: PLoS One. 2022 Oct 19;17(10):e0276224. doi: 10.1371/journal.pone.0276224 (PMC9581349; doi:10.1371/journal.pone.0276224)

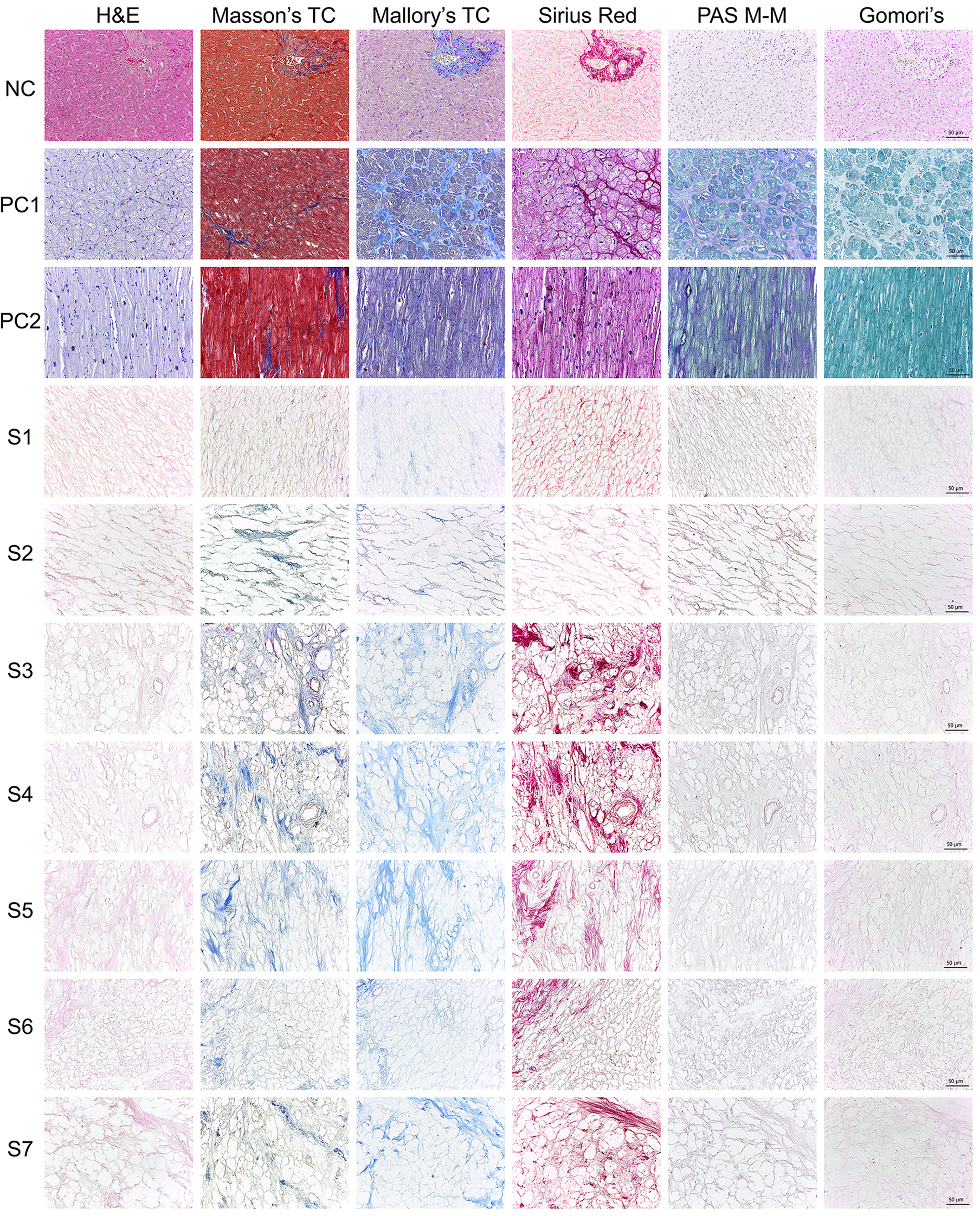

Supplement: S1 Fig — Representative images of Hematoxylin and Eosin, Masson’s and Mallory’s Trichrome, Sirius Red, PAS Morel-Maronger modified and Gomori’s paraldehyde-fuchsin stainings on cryosection sets of d-ECM (S1-S7) compared to liver sections as negative control (NC) and native heart sections as positive control (PC1-PC2). Scale bar length is 50 μm. (TIF) [file pone.0276224.s002.tif]

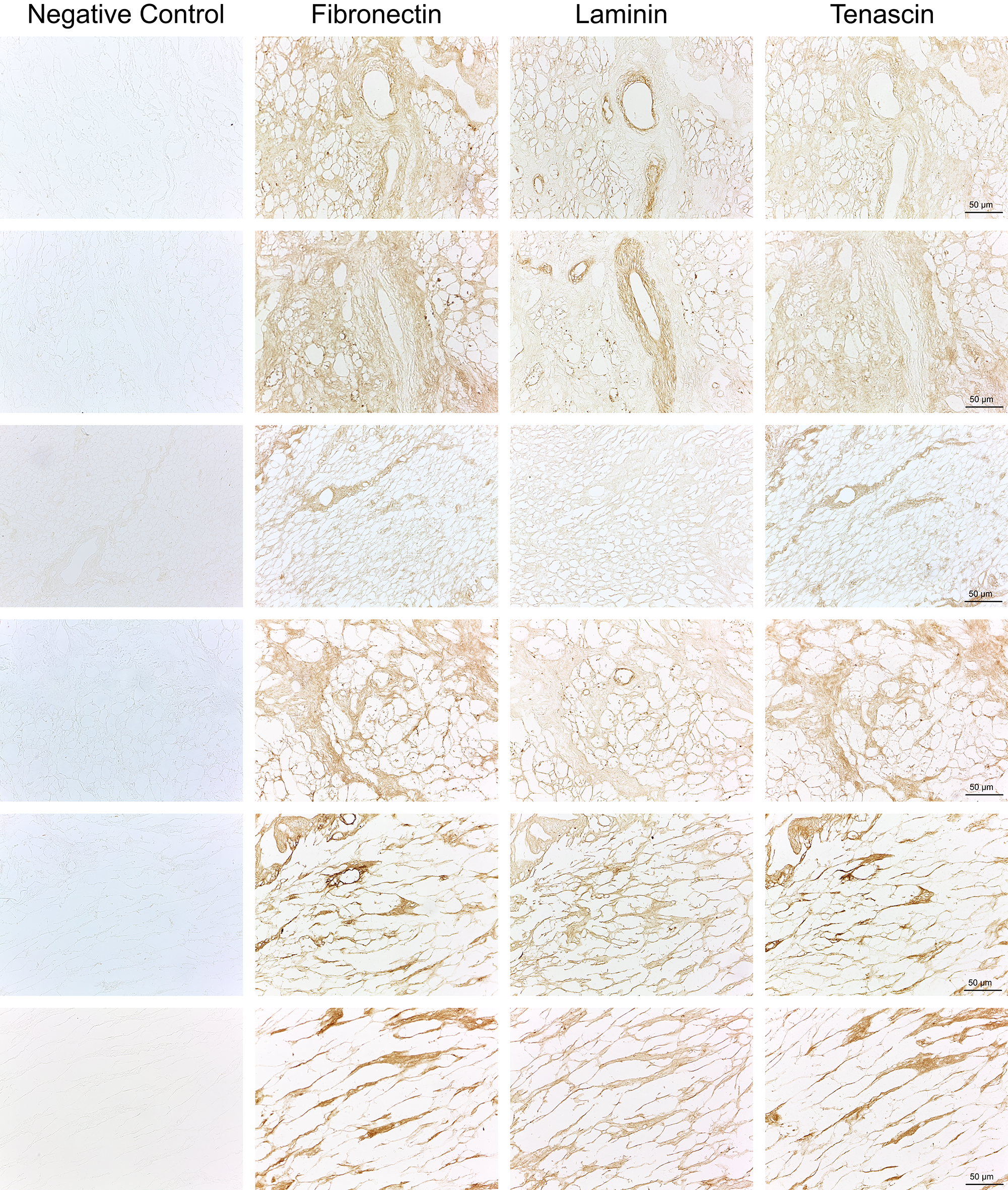

Supplement: S2 Fig — Representative images of fibronectin, laminin and tenascin distribution in cardiac d-ECM. Scale bar length is 50 μm. (TIF) [file pone.0276224.s003.tif]
