# Supplementary material for: Cardiac-derived extracellular matrix: A decellularization protocol for heart regeneration
Source: PLoS One. 2022 Oct 19;17(10):e0276224. doi: 10.1371/journal.pone.0276224 (PMC9581349; doi:10.1371/journal.pone.0276224)
Supplement: S1 Table — (DOCX) [file pone.0276224.s008.docx]

| **Gene symbol** | **Forward sequence** | **Reverse sequence** | **Amplicon**  **length (nt)** | |
| --- | --- | --- | --- | --- |
| GAPDH | 5’-CTCTCTGCTCCTCCTGTTCG-3’ | 5’-ACGACCAAATCCGTTGACTC-3’ | | 114 |
| MEF2C | 5’-AGGCAGCAAGAATACGATGC-3’ | 5’-TACGGAAACCACTGGGGTAG-3’ | | 88 |
| ACTC1 | 5’-TCGGGACCTCACTGACTACC-3’ | 5’-CAAAATCCAGGGCGACATAG-3’ | | 125 |
| ETS-1 | 5’-TGGGGACATCTTATGGGAAC-3’ | 5’-TGGATAGGCTGGGTTGACTC-3’ | | 88 |
| FVIII | 5’-GCTCTGGGATTATGGGATGA-3’ | 5’-TCTTGAACTGAGGGACACTGC-3’ | | 80 |
| GATA-6 | 5’-GTGTGCAATGCTTGTGGACT-3’ | 5’-TGTTCTTAGGTTTTCGTTTCCTG-3’ | | 103 |
| ACTA2 | 5’-CTGAGCGTGGCTATTCCTTC-3’ | 5’-TTCTCAAGGGAGGATGAGGA-3’ | | 133 |

**S8 Table** : Primers of genes analyzed by real-time PCR
