# Supplementary material for: Cardiac-derived extracellular matrix: A decellularization protocol for heart regeneration
Source: PLoS One. 2022 Oct 19;17(10):e0276224. doi: 10.1371/journal.pone.0276224 (PMC9581349; doi:10.1371/journal.pone.0276224)
Supplement: S1 Text — (DOCX) [file pone.0276224.s009.docx]

**Quantitative measurement of DNA content**

Genomic DNA (gDNA) was extracted from sections of frozen native myocardium and d-ECM, using the AllPrep DNA/RNA Mini Kit (Qiagen, Hilden, Germany), according to the

manufacturer’s instructions. DNA concentration was determined by measuring the absorbance at 260 nm using a Nanodrop1000 Spectrophotometer (Thermo Scientific, Waltham, MA, United States) and presented as ng DNA/mg of dry weight tissue. The DNA samples were size-fractionated by electrophoresis on 1% agarose gel with a potential of 90 V for 1 h in the TAE buffer (1×).

The DNA bands were visualized and photographed under UV light exposure with FireReader XS D-55 imaging system equipped with 1D software (UVItec Limited, Cambridge, UK).

**Quantitative measurement of collagen and sGAG**

For the evaluation of the residual collagen and sGAG content, Sircol or Blyscan quantitative dyebinding assays (Biocolor, Ltd, Carrickfergus, UK) were performed. To this end , a couple of native sections of equal weight was selected and one of them was decellularized to determine and compare collagen and sGAG content before and after the decellularizing procedure in the same sample. Each assay was performed at least in triplicate.

For the Sircol assay, 25 mg of each couple of sections were digested with the fragmentation reagent supplied by manufacturer, at 65°C for 2 hours. The digested samples were then assayed following the instructions supplied. The absorbance was read at 556 nm using the Sinergy H1 Hybrid Multi-Mode Reader (Agilent, CA, USA).

For the Blyscan assay, 30 mg of each couple of sections were digested using Papain Extraction Reagent supplied by the manufacturer, at 65°C for 3 hours. The digested samples were then assayed following the instructions supplied. The absorbance was read at 656 nm using the Sinergy H1 Hybrid Multi-Mode Reader (Agilent, CA, USA).

Data were averaged and expressed as mean values ± SEM of μg of collagen or sGAG per mg of wet tissue.

**Immunohistochemistry**

Decellularized sections were fixed in 10% neutral-buffered formalin, dehydrated in a graded series of alcohols, embedded in paraffin and then sliced into serial 5-µm-thick sections. For the immunodetection of fibronectin, laminin and tenascin, the obtained sections were deparaffinized, rehydrated, and immunostained using indirect immunoperoxidase technique and primary antibodies against human fibronectin, tenascin and laminin (all from Sigma‐Aldrich). The presence and localization of antigen antibody complexes were revealed by the UltraVision LP Detection System HRP Polymer & DAB Plus Chromogen (Thermo Scientific, Waltham, MA, United States). To assess that the results observed were due to the interaction between the target ECM proteins with the antibodies and not to false positives or non-specific bindings, samples incubated with just the diluent and without the primary antibody were used as negative control.

Stained sections were evaluated and documented by at least three independent observers using

a light microscope DM2000 Led (Leica Microsystems) equipped with an ICC50HD camera (Leica Microsystems).

**Real-time** **PCR**

Gene expression analysis was performed by Real-time quantitative assay. Total RNA was

extracted from hCPCs cultured for 21 days on d-ECM and on plastic dishes using Trizol Reagent (Invitrogen, Thermo Fisher Scientific, Carlsbad, CA, United States) according to the manufacturer's instructions. RNA was dissolved in RNase‐free water, and the final concentration was determined using the NanoDrop™ 1000 Spectrophotometer (Thermo Fisher Scientific, Jersey, New Jersey, USA). Then, the RNA from each sample was reverse transcribed into cDNA with the QuantiTect Reverse Transcription Kit (Qiagen), following the protocol provided by the supplier. Gene expression was analyzed by real‐time PCR using Power SYBR Green PCR Master Mix (Applied Biosystem, Thermo Fisher Scientific). DNA amplification was carried out on the Applied Biosystems 7500 Real-Time PCR system (Applied Biosystems, CA, USA) and the detection was performed by measuring the binding of the fluorescent dye SYBR Green I to double-stranded DNA.

The thermal cycling conditions included an initial enzyme activation at 95 °C for 2 min and 40 cycles consisting of a denaturation step at 95 °C for 15 s and an annealing step at 60 °C for 60 s.

The primers used were designed with Primer3 software (http://frodo.wi.mit.edu) starting from the coding sequence of mature mRNA and their sequences are detailed in S8 Table. Melt curve analyses were conducted to assess uniformity of product formation, primer dimer formation, and amplification of non‐specific products.

Comparative quantification of target genes expression in the samples was performed based on the cycle threshold (Ct) normalized to the housekeeping gene GAPDH using the ΔΔCt method [1]. Statistical analysis was performed using GraphPad Prism version 5.00 for Windows (GraphPad Software, San Diego, CA, United States2) and data were expressed as the mean ± SEM.

**References**

1. Livak KJ, Schmittgen TD. Analysis of relative gene expression data using real-time quantitative PCR and the 2(-Delta Delta C(T)) Method. Methods. 2001 Dec;25(4):402-8. doi: 10.1006/meth.2001.1262. PMID: 11846609.
